# Radiotherapy has a survival advantage over surgery in patients with choroidal melanoma: a retrospective cohort study of 6,871 patients

**DOI:** 10.3389/fsurg.2025.1577775

**Published:** 2025-04-01

**Authors:** Yifan Wu, Lu Shi, Zhiqiang Ye, Yi Zhou, Feiran Wang, Yulan Zhang

**Affiliations:** ^1^ Department of Ophthalmology, The Second Affiliated Hospital, Jiangxi Medical College, Nanchang University, Nanchang, Jiangxi, China; ^2^ Department of Ophthalmology, The Second Affiliated Hospital, Nanchang University, Nanchang, Jiangxi, China; ^3^ Department of General Practice, The Second Affiliated Hospital, Jiangxi Medical College, Nanchang University, Nanchang, Jiangxi, China

**Keywords:** choroidal melanoma, uveal melanoma, mortality, SEER database, treatment

## Abstract

**Background:**

Choroidal melanoma is a rare yet aggressive ocular malignancy, accounting for approximately 85% of all ocular melanomas. This study aimed to investigate the association between treatment modalities and the risk of all-cause mortality and choroidal melanoma-specific mortality, thereby comparing the effects of different treatment modalities on patient prognosis.

**Methods:**

Data from patients diagnosed with choroidal melanoma between 2004 and 2021 were extracted from the Surveillance, Epidemiology, and End Results (SEER) database. A total of 6,871 cases were included in the analysis. Univariate analysis, stratified analysis, and multiple regression analysis were performed to evaluate all-cause mortality and choroidal melanoma-specific mortality across different treatment modalities. Survival curves for the overall and stratified populations were generated using the Kaplan–Meier method. Choroidal melanoma-specific mortality was estimated using the competing risk regression method of Fine and Gray.

**Results:**

In the fully adjusted model, the radiotherapy-only group exhibited a 45% reduction in all-cause mortality (HR = 0.55, 95% CI = 0.50–0.60, *p* < 0.0001) and a 54% reduction in choroidal melanoma-specific mortality (HR = 0.46, 95% CI = 0.41–0.52, *p* < 0.0001) compared to the surgery-only group. The radiotherapy group demonstrated superior long-term survival outcomes compared to other treatment modalities, with the highest 5-year overall survival (OS) rate of 0.7769 (95% CI = 0.7651–0.7889) and 10-year OS rate of 0.6203 (95% CI = 0.6038–0.6372). Additionally, the radiotherapy group achieved the highest 5-year choroidal melanoma-specific survival (CSS) rate of 0.8615 (95% CI = 0.8514–0.8717) and 10-year CSS rate of 0.7715 (95% CI = 0.7567–0.7866).

**Conclusions:**

Among patients diagnosed with choroidal melanoma, those who underwent radiotherapy alone exhibited significantly higher overall survival (OS) and choroidal melanoma-specific survival rates compared to those who received surgical intervention alone. However, for patients with advanced disease or evidence of metastatic spread, the individualization of treatment regimens remains critically important.

## Introduction

Choroidal melanoma, the most prevalent subtype of uveal melanoma, accounts for approximately 85% of all ocular melanoma cases (1). Despite its relatively low overall incidence (age-standardized incidence rate of 5.1 cases per million in the United States) (2), choroidal melanoma is recognized as one of the most lethal ocular malignancies due to its high metastatic potential. The 15-year cumulative metastasis rate is approximately 49% (3), with the liver being the most common site of metastasis, followed by the breast and lungs (4). Epidemiological studies have demonstrated significant geographic and ethnic variations in disease incidence. The highest rates are observed in Northern European countries, such as Denmark (8.6 cases per million), while the lowest rates are found in Asian populations, particularly in Japan (0.3 cases per million) (5). Furthermore, a distinct gender disparity exists, with male patients exhibiting both higher incidence rates (5.8 vs. 4.4 cases per million) and poorer prognosis compared to their female counterparts (6).

The management strategies for choroidal melanoma have undergone significant evolution over the past decades. Historically, ocular enucleation served as the primary treatment modality; however, recent advancements have established eye-preserving therapies, particularly I-125 episcleral plaque brachytherapy and transpupillary thermotherapy, as the preferred treatment options for small- to medium-sized tumors (7).

The superiority of radiotherapy lies in its ability to achieve effective tumor control (10-year local control rate >90%) while preserving both ocular function and appearance (8). Despite the excellent local control achieved by eye-preserving therapies, their impact on long-term survival remains controversial. For large tumors (thickness >8 mm or basal diameter >16 mm), enucleation remains the primary option due to the potential for severe visual acuity reduction and dry eye syndrome associated with radiotherapy (9). The Collaborative Ocular Melanoma Study (COMS) conducted a 12-year follow-up investigation of 1,317 patients and found no significant difference in the all-cause mortality between I-125 brachytherapy and enucleation (HR = 1.07, 95% CI 0.88–1.30) (10). Additionally, surgical enucleation may potentially facilitate circulating tumor cell (CTC) dissemination through mechanical manipulation, whereas radiotherapy-induced immunogenic cell death (ICD) might suppress the development of micrometastases (11). Although the COMS study has provided critical evidence for treatment selection, its findings are primarily derived from patients with small- to medium-sized tumors. Furthermore, the majority of existing studies are single-center retrospective analyses with limited sample sizes (typically <500 cases) and insufficient long-term follow-up data (12). These limitations hinder the ability of current evidence to support individualized treatment decisions, particularly in the context of emerging therapies such as immunotherapy and targeted treatments (13).

To address these limitations, this study extracted data from the Surveillance, Epidemiology, and End Results (SEER) database of the National Cancer Institute, which collects cancer diagnosis, treatment, and survival data for approximately 30% of the US population. Based on data from the SEER 9 registry between 2004 and 2021, we examined the relationship between treatment modalities and both all-cause and choroidal melanoma-specific mortality in 6,871 patients, adjusting for age, gender, race/ethnicity, tumor stage, tumor size, tumor laterality, International Classification of Diseases for Oncology, Third Edition (ICD-O-3) histology/behavior, and year of diagnosis. Our study directly compared the impact of three treatment modalities—surgery alone (including enucleation, local tumor resection, etc.), radiotherapy alone, and combined surgery with radiotherapy—on both all-cause mortality and choroidal melanoma-specific mortality.

## Materials and methods

### Data source and patient selection

We extracted data from the most recent SEER 9 Registry Research database (submitted November 2023), which covers the period from 2004 to 2021. The SEER 9 database comprises research data from 17 registries, representing approximately 10% of the U.S. population. The reliability of the findings is ensured by the database's extensive coverage and rigorous methodology. This study complies with the NCI SEER limited-use data end-user agreement. As all data used in this study are publicly available, no institutional review board approval was required.

### Cohort selection

**Figure 1:**
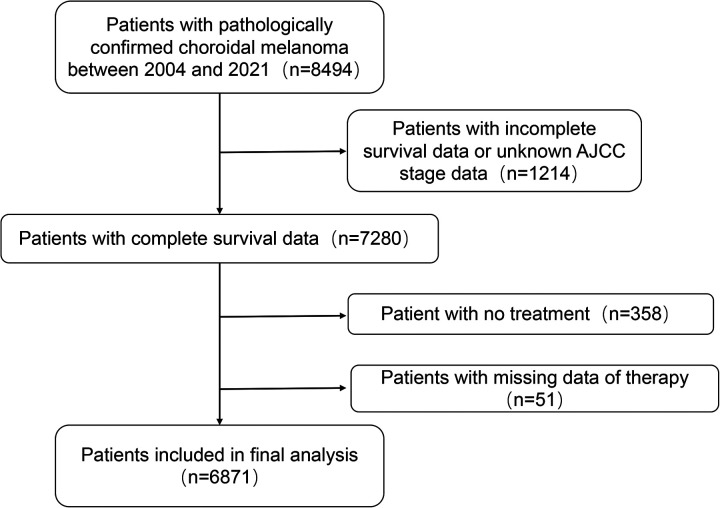
Flow chart of cases selection from SEER database.

SEER*Stat version 8.3.9.2 (seer.cancer.gov/seerstat) was used to generate the case list. Cases were extracted from patients diagnosed with choroidal melanoma over the past 20 years. The case list included the following variables: age, race, sex, year of diagnosis, primary site, laterality, histology, T stage, N stage, M stage, treatment, and marital status. Race was categorized as White and Others. Treatment data were also extracted, including surgery (yes/no) and combined radiotherapy (yes/no). Age was stratified into four brackets: 0–25 years, 26–50 years, 51–75 years, and 75+ years. Choroidal melanoma was classified into three grades according to the Collaborative Ocular Melanoma Study (COMS): large, medium, and small. The primary site and morphology for choroidal melanoma patients were selected using the “Site recode ICD-O-3/WHO 2008” variable, with “C69.3-Choroid” as the primary labeled site. Initial inclusion of all choroidal cancer cases yielded 8,494 cases, encompassing the following histologic subtypes: 8720/3 (Malignant melanoma, NOS), 8721/3 (Nodular melanoma), 8722/3 (Balloon cell melanoma), 8723/3 (Malignant melanoma, regressing), 8730/3 (Amelanotic melanoma), 8740/3 (Malignant melanoma in junctional nevus), 8743/3 (Superficial spreading melanoma), 8745/3 (Desmoplastic melanoma, malignant), 8761/3 (Malignant melanoma in giant pigmented nevus), 8770/3 (Mixed epithelioid and spindle cell melanoma), 8771/3 (Epithelioid cell melanoma), 8772/3 (Spindle cell melanoma, NOS), 8773/3 (Spindle cell melanoma, type A), and 8774/3 (Spindle cell melanoma, type B). We excluded 1,214 patients with incomplete survival or AJCC stage data, 358 cases that underwent no treatment, and 51 cases with missing treatment modality data. Ultimately, 6,871 cases were included in the study. The flow chart of the patient selection process is presented in Figure 1.

### Vital status

The status of the patients at the most recent follow-up was extracted using SEER 9's “cause of death (COD) to site recode” variable. Based on these data, patients were categorized into three groups: (1) patients who survived, (2) patients who died from choroidal melanoma, and (3) patients who died from other causes. The primary outcome was all-cause mortality, while the secondary outcomes included choroidal melanoma-specific mortality and non-choroidal melanoma-specific mortality. Temporal information, from the date of diagnosis to the date of the last follow-up, was extracted using the variable “survival months.” The SEER*Stat program calculated survival time (in months) by subtracting the date of diagnosis from the date of last contact (study cut-off date: December 31, 2021).

### Statistical analysis

Patients were categorized based on the types of therapies they received, including the surgery-only group and the surgery plus radiation group. Overall survival (OS) was defined as the time interval from the diagnosis of choroidal melanoma to death from any cause, while choroidal melanoma-specific survival (CSS) was defined as the time interval from diagnosis to death specifically attributed to choroidal melanoma. Univariate analysis (unadjusted) was performed to identify covariates associated with mortality, and stratified analysis (adjusted) was conducted to evaluate the impact of each population subgroup on mortality. The Kaplan–Meier (KM) method was used to plot survival curves according to different treatment modalities. KM curves for all-cause survival and choroidal melanoma-specific survival, stratified by variables such as sex, were generated to assess the effect of treatment modality on patient survival across different populations. Cox proportional hazards regression analysis was employed to examine the effects of age, race, sex, year of diagnosis, laterality, histology, treatment, marital status, T stage, N stage, and M stage on all-cause mortality and choroidal melanoma-specific mortality in patients with choroidal melanoma. (The TNM staging system characterizes tumor biology through standardized evaluation of three components: primary tumor dimensions, regional lymph node status, and presence of distant metastases.) The competing risk regression method of Fine and Gray was used to estimate choroidal melanoma-specific mortality. EmpowerStats, a statistical software based on the R language, was utilized for data analysis. This software offers robust data processing capabilities and comprehensive analytical functions. The agreed cut-off for statistical significance was *p* < 0.05.

## Results

### Baseline characteristics of the study participants by treatment modality

A total of 6,871 patients diagnosed with choroidal melanoma between 2004 and 2021 were identified from the SEER database and included in the analysis. The cohort was stratified by age as follows: 83 patients (1.2%) aged 0–25 years, 1,128 patients (16.4%) aged 26–50 years, 4,299 patients (62.6%) aged 51–75 years, and 1,361 patients (19.8%) aged 75 years or older at the time of diagnosis. Among the patients, 3,607 (52.5%) were male, and 3,264 (47.5%) were female. Regarding treatment modalities, 20.3% of the patients underwent surgery only, 71.4% received radiotherapy only, and 8.3% received surgery combined with radiotherapy. A strong correlation was observed between treatment modality and tumor stage: T1 and T2 stages were present in 32.24% and 36.62% of patients receiving radiotherapy only, and in 36.80% and 31.69% of patients in the surgery plus radiation group, respectively. However, no significant difference in tumor stages was observed among patients in the surgery-only group. The baseline characteristics of the study population are presented in Table 1.

**Table 1:** Baseline characteristics of the study participants by treatment method.

Treatment	Surgery only	Radiation only	Surgery + radiation	*P*-value
Age				0.006
0–25 years	25 (1.79%)	54 (1.10%)	4 (0.70%)	
26–50 years	240 (17.19%)	788 (16.06%)	100 (17.61%)	
51–75 years	817 (58.52%)	3,122 (63.62%)	360 (63.38%)	
75+ years	314 (22.49%)	943 (19.22%)	104 (18.31%)	
Sex				<0.001
Male	805 (57.66%)	2,497 (50.89%)	305 (53.70%)	
Female	591 (42.34%)	2,410 (49.11%)	263 (46.30%)	
Year of diagnosis				0.231
2004–2014	819 (58.67%)	2,753 (56.10%)	320 (56.34%)	
2015–2021	577 (41.33%)	2,154 (43.90%)	248 (43.66%)	
Race				0.002
White	1,326 (94.99%)	4,755 (96.90%)	552 (97.18%)	
Other/Unknown	70 (5.01%)	152 (3.10%)	16 (2.82%)	
Laterality				0.096
Left	686 (49.14%)	2,477 (50.48%)	267 (47.01%)	
Right	708 (50.72%)	2,424 (49.40%)	298 (52.46%)	
Unknown	2 (0.14%)	6 (0.12%)	3 (0.53%)	
Tumor size				<0.001
Small	89 (6.38%)	472 (9.62%)	69 (12.15%)	
Medium	141 (10.10%)	502 (10.23%)	64 (11.27%)	
Large	122 (8.74%)	172 (3.51%)	29 (5.11%)	
Unrecorded	1,044 (74.79%)	3,761 (76.65%)	406 (71.48%)	
Marital status				<0.001
Married	723 (51.79%)	3,157 (64.34%)	361 (63.56%)	
Divorced	113 (8.09%)	356 (7.25%)	50 (8.80%)	
Single	230 (16.48%)	653 (13.31%)	65 (11.44%)	
Other	330 (23.64%)	741 (15.10%)	92 (16.20%)	
Stage T				<0.001
T1	286 (20.49%)	1,582 (32.24%)	209 (36.80%)	
T2	305 (21.85%)	1,797 (36.62%)	180 (31.69%)	
T3	404 (28.94%)	593 (12.08%)	69 (12.15%)	
T4	199 (14.26%)	89 (1.81%)	27 (4.75%)	
Tx	202 (14.47%)	846 (17.24%)	83 (14.61%)	
Stage N				<0.001
N0	1,263 (90.47%)	4,624 (94.23%)	540 (95.07%)	
N1	2 (0.14%)	7 (0.14%)	1 (0.18%)	
Nx	131 (9.38%)	276 (5.62%)	27 (4.75%)	
Stage M				<0.001
M0	1,305 (93.48%)	4,720 (96.19%)	552 (97.18%)	
M1	40 (2.87%)	46 (0.94%)	5 (0.88%)	
Mx	51 (3.65%)	141 (2.87%)	11 (1.94%)	

Note: Continuous variables were presented as mean ± SD; Categorical variables were presented as *n* (%).

### Univariate analysis of the association between treatment modality and mortality

In the unadjusted univariate analysis (Table 2), all-cause mortality was significantly lower in the radiation-only group [hazard ratio [HR] = 0.45, 95% confidence interval [CI] = 0.41–0.50, *p* < 0.0001] and the surgery plus radiation group (HR = 0.58, 95% CI = 0.49–0.68, *p* < 0.0001) compared with the surgery-only group. Similarly, choroidal melanoma-specific mortality was significantly reduced in the radiation-only group (HR = 0.37, 95% CI = 0.33–0.41, *p* < 0.0001) and the surgery plus radiation group (HR = 0.57, 95% CI = 0.47–0.70, *p* < 0.0001) relative to the surgery-only group. Tumor size had a significant impact on survival rates. Specifically, all-cause mortality was higher in patients with medium-sized tumors (HR = 1.76, 95% CI = 1.48–2.08, *p* < 0.0001) and large-sized tumors (HR = 2.94, 95% CI = 2.44–3.54, *p* < 0.0001) compared with those with small-sized tumors. Choroidal melanoma-specific mortality also increased significantly in patients with medium-sized tumors (HR = 3.27, 95% CI = 2.50–4.27, *p* < 0.0001) and large-sized tumors (HR = 6.15, 95% CI = 4.64–8.15, *p* < 0.0001) compared with those with small-sized tumors. Marital status influenced survival outcomes. Divorced patients exhibited higher all-cause mortality (HR = 1.24, 95% CI = 1.07–1.43, *p* = 0.0053) and choroidal melanoma-specific mortality (HR = 1.26, 95% CI = 1.04–1.51, *p* = 0.0167) compared with the surgery-only population. Both all-cause mortality and choroidal melanoma-specific mortality were associated with multiple clinical factors. All-cause mortality was related to age, sex, tumor size, marital status, T stage, N stage, and M stage. Choroidal melanoma-specific mortality was associated with year of diagnosis, tumor size, marital status, T stage, N stage, and M stage.

**Table 2:** Crude univariate analysis of the association between treatment method and mortality.

Statistics	Vital status	Hazard ratio (95% CI) *p*-value	
		All-cause mortality	Choroidal melanoma-specific mortality
Age			
0–25 years	83 (1.21%)	1	1
26–50 years	1,128 (16.42%)	0.73 (0.45, 1.20) 0.2188	0.66 (0.40, 1.09) 0.1066
51–75 years	4,299 (62.57%)	1.56 (0.97, 2.52) 0.0673	1.05 (0.65, 1.70) 0.8381
75+ years	1,361 (19.81%)	4.06 (2.51, 6.56) < 0.0001	1.54 (0.94, 2.50) 0.0845
Sex			
Male	3,607 (52.50%)	1	1
Female	3,264 (47.50%)	0.91 (0.84, 0.98) 0.0197	0.99 (0.89, 1.10) 0.8914
Year of diagnosis			
2004–2014	3,892 (56.64%)	1	1
2015–2021	2,979 (43.36%)	0.90 (0.81, 1.00) 0.0555	0.86 (0.76, 0.99) 0.0324
Race			
White	6,633 (96.54%)	1	1
Other/Unknown	238 (3.46%)	0.85 (0.67, 1.08) 0.1927	1.07 (0.81, 1.41) 0.6417
Treatment			
Surgery only	1,396 (20.32%)	1	1
Radiation only	4,907 (71.42%)	0.45 (0.41, 0.50) < 0.0001	0.37 (0.33, 0.41) < 0.0001
Surgery + Radiation	568 (8.27%)	0.58 (0.49, 0.68) < 0.0001	0.57 (0.47, 0.70) < 0.0001
Laterality			
Left	3,430 (49.92%)	1	1
Right	3,430 (49.92%)	1.02 (0.94, 1.10) 0.6706	1.02 (0.92, 1.13) 0.7373
Unknown	11 (0.16%)	1.84 (0.92, 3.68) 0.0864	2.46 (1.10, 5.49) 0.0283
Tumor size			
Small	630 (9.17%)	1.0	1.0
Medium	707 (10.29%)	1.76 (1.48, 2.08) < 0.0001	3.27 (2.50, 4.27) < 0.0001
Large	323 (4.70%)	2.94 (2.44, 3.54) < 0.0001	6.15 (4.64, 8.15) < 0.0001
Unrecorded	5,211 (75.84%)	1.57 (1.36, 1.81) < 0.0001	2.71 (2.13, 3.46) < 0.0001
Marital status			
Married	4,241 (61.72%)	1	1
Divorced	519 (7.55%)	1.24 (1.07, 1.43) 0.0053	1.26 (1.04, 1.51) 0.0167
Single	948 (13.80%)	1.06 (0.93, 1.20) 0.3973	0.94 (0.80, 1.11) 0.4741
Other	1,163 (16.93%)	1.58 (1.43, 1.75) < 0.0001	1.27 (1.10, 1.46) 0.0009
Stage T			
T1	2,077 (30.23%)	1	1
T2	2,282 (33.21%)	1.48 (1.33, 1.64) < 0.0001	1.82 (1.56, 2.12) < 0.0001
T3	1,066 (15.51%)	2.72 (2.41, 3.08) < 0.0001	4.14 (3.52, 4.87) < 0.0001
T4	315 (4.58%)	4.33 (3.58, 5.22) < 0.0001	6.60 (5.23, 8.34) < 0.0001
Tx	1,131 (16.46%)	1.71 (1.49, 1.97) < 0.0001	2.20 (1.82, 2.66) < 0.0001
Stage N			
N0	6,427 (93.54%)	1	1
N1	10 (0.15%)	2.20 (1.05, 4.62) 0.0372	2.62 (1.09, 6.31) 0.0314
Nx	434 (6.32%)	1.17 (1.00, 1.37) 0.0560	1.30 (1.07, 1.58) 0.0084
Stage M			
M0	6,577 (95.72%)	1	1
M1	91 (1.32%)	6.35 (5.04, 8.00) < 0.0001	9.20 (7.16, 11.81) < 0.0001
Mx	203 (2.95%)	1.09 (0.88, 1.35) 0.4207	1.23 (0.94, 1.60) 0.1338

### Multivariate analysis of the association between treatment modality and mortality

In the multiple regression analysis, both all-cause mortality and choroidal melanoma-specific mortality were significantly lower in the radiation-only group compared with the other groups (Table 3). In the analysis with all-cause mortality as the outcome variable, the groups were ranked by mortality rates from lowest to highest, in ascending order: the radiation-only group, the surgery plus radiation group, and the surgery-only group. This ranking was consistent across the unadjusted model and models I and II, which were adjusted for sociodemographic and clinical variables. In the fully adjusted model, all-cause mortality was 45% lower in the radiation-only group (HR = 0.55, 95% CI = 0.50–0.60, *p* < 0.0001) and 27% lower in the surgery plus radiation group (HR = 0.73, 95% CI = 0.63–0.86, *p* < 0.0001) compared with the surgery-only group.

**Table 3:** Multivariate analysis of the association between treatment method and mortality.

Exposure	Hazard ratio (95% CI) *p*-value		
	Non-adjusted	Adjust I	Adjust II
All-cause mortality			
Treatment			
Surgery only	1	1	1
Radiation only	0.45 (0.41, 0.50) < 0.0001	0.45 (0.41, 0.49) < 0.0001	0.55 (0.50, 0.60) < 0.0001
Surgery + radiation	0.58 (0.49, 0.68) < 0.0001	0.58 (0.50, 0.68) < 0.0001	0.73 (0.63, 0.86) < 0.0001
Choroidal melanoma-specific mortality			
Treatment			
Surgery only	1	1	1
Radiation only	0.37 (0.33, 0.41) < 0.0001	0.36 (0.32, 0.40) < 0.0001	0.46 (0.41, 0.52) < 0.0001
Surgery + radiation	0.37 (0.33, 0.41) < 0.0001	0.57 (0.47, 0.70) < 0.0001	0.76 (0.63, 0.93) 0.0067

Note: Non-adjusted model adjusted for: None. Adjusted I model adjusted for: Age; Race; Sex. Adjusted II model adjusted for: Age; Race; Sex; Year of diagnosis; Primary site; Laterality; Histology; T stage; N stage; M stage; Marital status.

Similarly, in the analysis of choroidal melanoma-specific mortality as the outcome variable, the groups were ranked by mortality rates from lowest to highest, in ascending order: the radiation-only group, the surgery plus radiation group, and the surgery-only group. In the fully adjusted model, choroidal melanoma-specific mortality was 54% lower in the radiation-only group (HR = 0.46, 95% CI = 0.41–0.52, *p* < 0.0001) and 24% lower in the surgery plus radiation group (HR = 0.76, 95% CI = 0.63–0.93, *p* = 0.0067) compared with the surgery-only group. The specific adjustment variables are detailed in Table 3.

### Overall survival and choroidal melanoma cancer-specific survival for people with different treatment modalities

**Figure 2:**
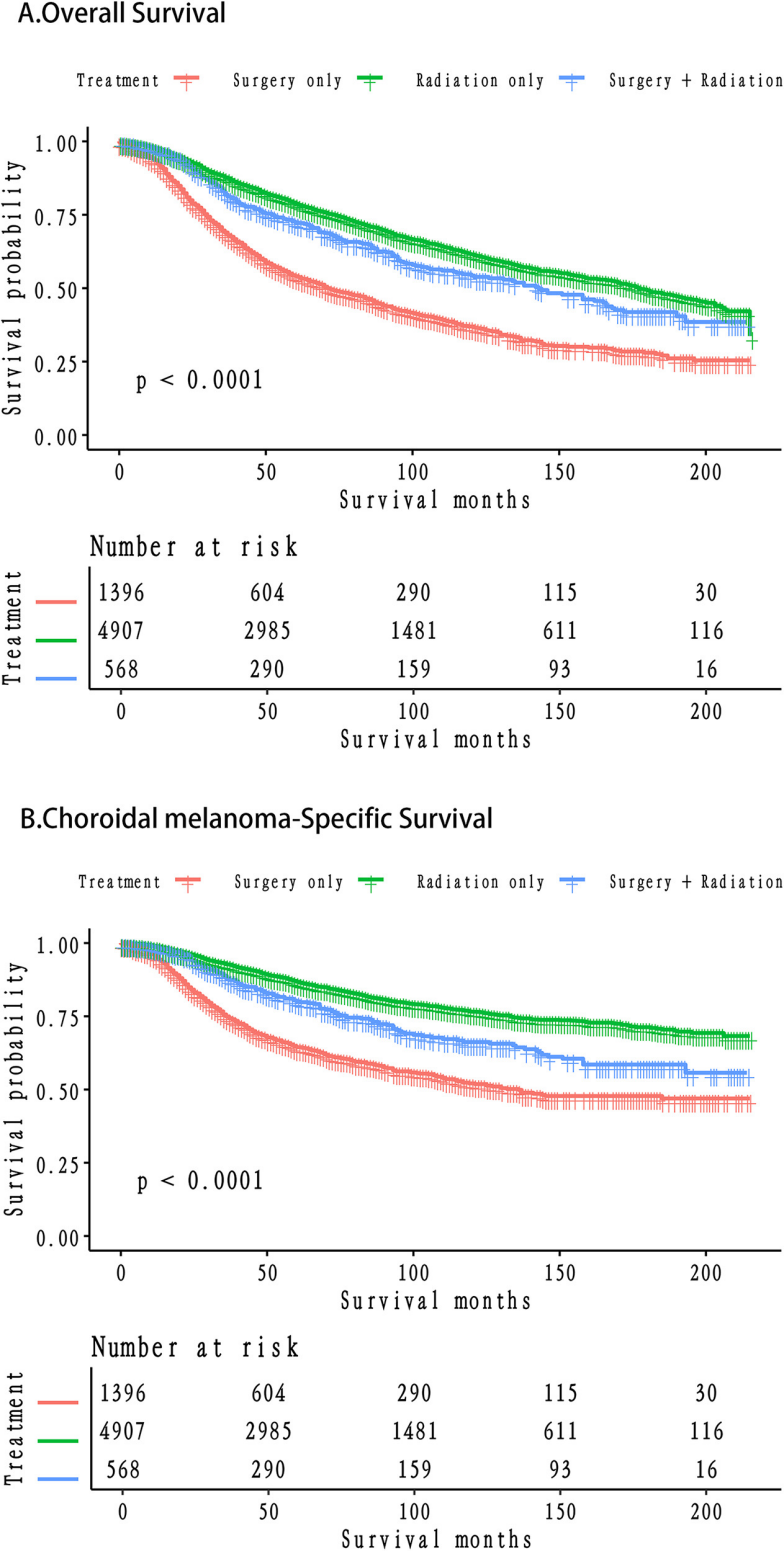
Survival stratified by treatment modalities among patients **(A)** Overall survival; **(B)** Choroidal melanoma-specific survival.

The radiation-only group exhibited the highest overall survival (OS) rates (Table 4; Figure 2A). Specifically, the 5-year OS rates were 0.5719 (95% CI = 0.547–0.5978), 0.7769 (95% CI = 0.7651–0.7889), and 0.7244 (95% CI = 0.6921–0.7582) in the surgery-only, radiation-only, and surgery plus radiation groups, respectively. The corresponding 10-year OS rates were 0.3474 (95% CI = 0.3199–0.3771), 0.6203 (95% CI = 0.6038–0.6372), and 0.5433 (95% CI = 0.4990–0.5917), while the 15-year OS rates were 0.2261 (95% CI = 0.1999–0.2556), 0.5109 (95% CI = 0.4899–0.5328), and 0.4241 (95% CI = 0.3758–0.4786), respectively.

**Table 4:** Overall survival and choroidal melanoma-specific survival for people treated using different methods.

Treatment	Surgery only	Radiation only	Surgery + radiation
Overall survival			
*N*	1,396	4,907	568
5-year survival (95% CI)	57.19% (54.70%–59.78%)	77.69% (76.51%–78.89%)	72.44% (69.21%–75.82%)
10-year survival (95% CI)	34.74% (31.99%–37.71%)	62.03% (60.38%–63.72%)	54.33% (49.90%–59.17%)
15-year survival (95% CI)	22.61% (19.99%–25.56%)	51.09% (48.99%–53.28%)	42.41% (37.58%–47.86%)
Choroidal melanoma-special survival			
*N*	1,396	4,907	568
5-year survival (95% CI)	66.50% (63.95%–69.16%)	86.15% (85.14%–87.17%)	79.14% (75.98%–82.43%)
10-year survival (95% CI)	49.18% (46.00%–52.59%)	77.15% (75.67%–78.66%)	66.56% (62.07%–71.39%)
15-year survival (95% CI)	41.52% (38.04%–45.32%)	72.52% (70.66%–74.42%)	60.40% (55.34%–65.92%)

Note: CI, confidence interval.

Similarly, the radiation-only group demonstrated the highest choroidal melanoma-specific survival rates (Table 4; Figure 2B). The 5-year choroidal melanoma-specific survival rates were 0.6650 (95% CI = 0.6395–0.6916), 0.8615 (95% CI = 0.8514–0.8717), and 0.7914 (95% CI = 0.7598–0.8243) in the surgery-only, radiation-only, and surgery plus radiation groups, respectively. The corresponding 10-year survival rates were 0.4918 (95% CI = 0.46–0.5259), 0.7715 (95% CI = 0.7567–0.7866), and 0.6656 (95% CI = 0.6207–0.7139), while the 15-year survival rates were 0.4152 (95% CI = 0.3804–0.4532), 0.7252 (95% CI = 0.7066–0.7442), and 0.604 (95% CI = 0.5534–0.6592), respectively. To further analyze the survival of patients receiving different treatment modalities in different populations, we plotted stratified KM curves.

**Figure 3:**
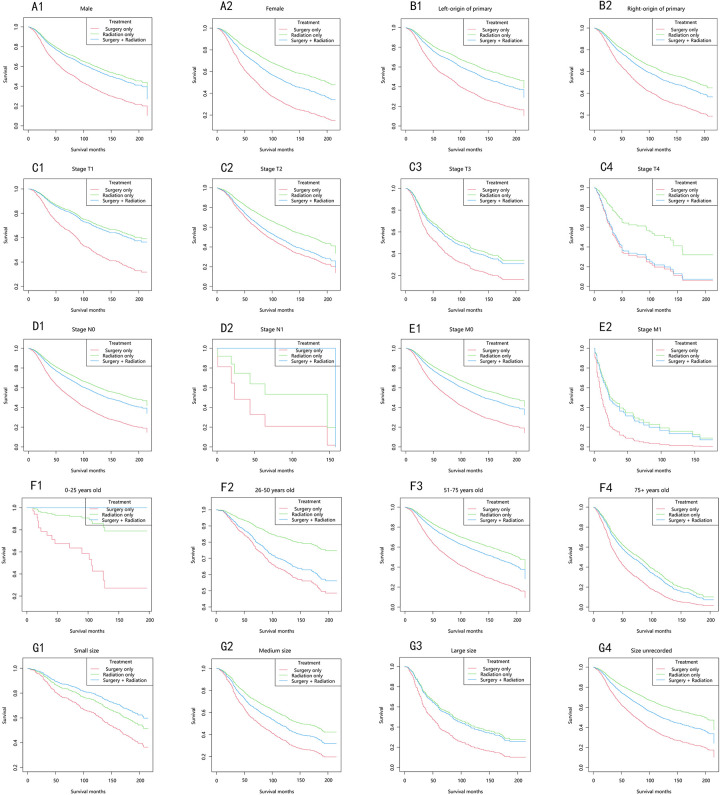
Overall survival stratified by treatment modalities among patients with choroidal melanoma in different stratifications. **(A1–A2)** Stratified by sex; **(B1–B2)** stratified by laterality; **(C1–C4)** stratified by stage T; **(D1–D2)** stratified by stage N; **(E1–E2)** stratified by stage M; **(F1–F4)** stratified by years; **(G1–G4)** stratified by size.

The longest overall survival (OS) was observed in the majority of strata, in descending order: the radiation-only group, the surgery-plus-radiation group, and the surgery-only group (Figure 3). Among patients with stage T1, T3, M1, and large tumor size, the OS curves in the surgery-plus-radiation group nearly overlapped with those in the radiation-only group. In patients with stage T4, the OS curves in the surgery-plus-radiation group overlapped with those in the surgery-only group. Among patients staged as N1, aged 0–25 years, or with small tumor size, the surgery-plus-radiation group exhibited the highest survival rate.

**Figure 4:**
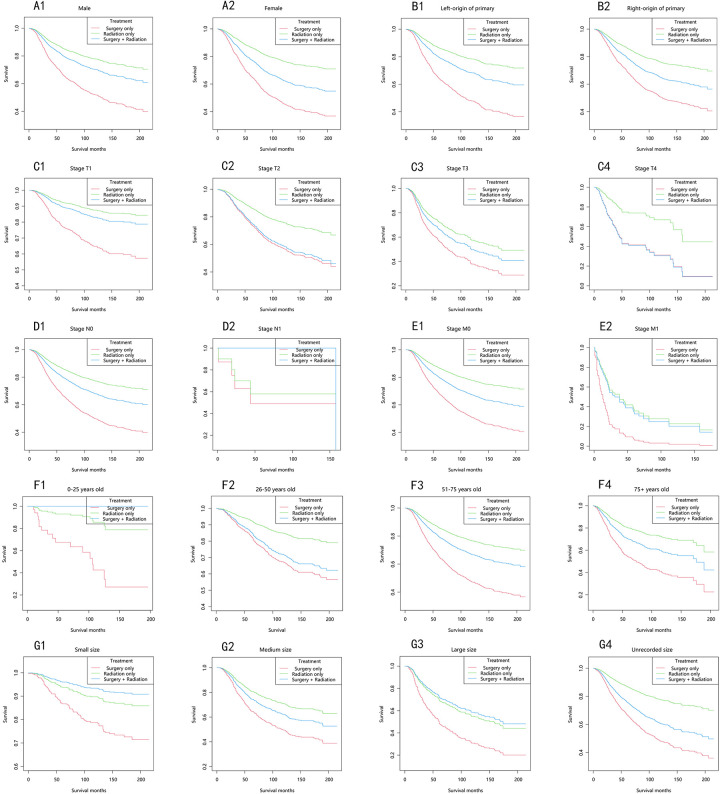
Choroidal melanoma-specific survival stratified by treatment modalities among patients with choroidal melanoma in different stratifications. **(A1–A2)** Stratified by sex; **(B1–B2)** stratified by laterality; **(C1–C4)** stratified by stage T; **(D1–D2)** stratified by stage N; **(E1–E2)** stratified by stage M; **(F1–F4)** stratified by years; **(G1–G4)** stratified by size.

In the majority of patients, the choroidal melanoma-specific survival rates followed a similar trend, in descending order: the radiation-only group, the surgery-plus-radiation group, and the surgery-only group (Figure 4). Among patients with stage T2 and T4, the choroidal melanoma-specific survival curves in the surgery-plus-radiation group nearly overlapped with those in the surgery-only group. In patients with stage M1 and large tumor size, the choroidal melanoma-specific survival curves in the surgery-plus-radiation group overlapped with those in the radiation-only group. Among patients staged as N1, aged 0–25 years, or with small tumor size, the surgery-plus-radiation group demonstrated the highest survival rate.

### Competing risk model analysis of the relationship between treatment modality and mortality

In the adjusted competing risks model, there was a significant difference in the risk of death among patients receiving different treatment modalities (Table 5). In the analysis of non-choroidal melanoma death as an outcome indicator. Using the surgery-only group as a reference, the risk of death in the radiation-only group and the surgery plus radiation group were 0.45 (95% CI = 0.41–0.49, *p* < 0.0001) and 0.58 (95% CI = 0.50–0.68, *p* < 0.0001), respectively. In the analysis using death from choroidal melanoma as the outcome indicator, the risk of death from choroidal melanoma were 0.36 (95% CI = 0.32–0.40, *p* < 0.0001) and 0.57 (95% CI = 0.47–0.69, *p* < 0.0001) in the radiation-only group and the surgery plus radiation group respectively, compared with the surgery-only group. The specific adjustment variables are detailed in Table 5.

**Table 5:** Treatment and cause-specific mortality in the cohort.

Treatment	Death (not attributable to Choroidal melanoma)		Death (attributable to Choroidal melanoma)	
	Deaths (*N*)	HR (95%) CI *p*-value	Deaths (*N*)	HR (95% CI) *p*-value
Surgery only	232	1 (Ref)	482	1 (Ref)
Radiation only	667	0.45 (0.41–0.49) *p* < 0.0001	790	0.36 (0.32–0.40) *p* < 0.0001
Surgery + radiation	70	0.58 (0.50–0.68) *p* < 0.0001	134	0.57 (0.47–0.69) *p* < 0.0001

Note: HR, hazard ratio; CI, confidence interval. Adjusted by age; race; sex; year of diagnosis; primary site; laterality; histology; T stage; N stage; M stage; marital status.

## Discussion

Choroidal melanoma, a rare yet aggressive ocular malignancy, accounts for 85% of all ocular melanoma cases. As the first large-scale cohort study (*n* = 6,871) utilizing the SEER database (2004–2021) to compare the three primary treatment modalities for choroidal melanoma, this study demonstrated that radiotherapy significantly improved both 5-year overall survival (OS) (77.7% vs. 57.2%) and 10-year cancer-specific survival (CSS) (77.1% vs. 49.2%) compared to surgery alone. These results provide a novel perspective on the traditional surgery-dominated treatment paradigm and offer new evidence to inform updates to the AJCC guidelines. Our findings are consistent with the Collaborative Ocular Melanoma Study (COMS), which, after 12 years of follow-up, reported no significant survival benefit of enucleation over radiotherapy (14). Similarly, Jang et al. used propensity score matching and found that the 5-year overall survival (OS) and cancer-specific survival (CSS) were significantly higher in the radiotherapy group compared to the surgery group (76% vs. 60% for OS; 89% vs. 73% for CSS). This finding demonstrates that radiotherapy yields better survival rates than surgery, particularly for patients with early T-stage disease (2). We conducted stratified analyses of various variables, and the results demonstrated that age, gender, marital status, and T-stage significantly impact patient prognosis. In both adjusted and unadjusted models, advancing age was significantly associated with an increased risk of both all-cause mortality and cancer-specific mortality. Maria et al. (15) identified significant differences in clinical characteristics and prognosis among uveal melanoma (UM) patients of different ages. They observed that age-related survival disparities may reflect a combination of factors, including later-stage diagnosis, increased metastatic potential, and reduced disease resistance due to age-related comorbidities in older patients.

To explore the impact of gender on patient survival, Maria et al. found that male patients were more prevalent and had worse prognoses compared to female patients (15). In our study, the all-cause mortality rate was significantly lower for females than for males (*P* = 0.02), although no significant difference in choroidal melanoma-specific mortality was observed between the two gender (*P* = 0.89). We hypothesize that male patients may be more susceptible to earlier mortality from underlying conditions, such as cardiovascular and pulmonary diseases (16), which could contribute to the higher all-cause mortality rate. Additionally, Feyza et al. (17) noted in their study on conjunctival melanoma that male gender is an independent risk factor for tumor metastasis, further supporting our findings. Our results also indicate that race is not a significant risk factor for patient prognosis, likely due to the predominance of White patients in the study population.

Marital status is a significant factor influencing psychological well-being, and previous studies have demonstrated its substantial impact on the prognosis of various malignancies, including lung, colorectal, breast, and pancreatic cancers.Unmarried patients (including widowed individuals) exhibit a higher risk of cancer metastasis, inadequate treatment, and mortality compared to their married counterparts (18). Our study similarly found that married patients with choroidal melanoma had better prognoses than divorced or single patients. These findings align with those of Libby Ellis et al. (2018), who demonstrated that married cancer patients experience superior survival outcomes, potentially attributable to the emotional and financial support provided by spouses, which may enhance treatment adherence and access to high-quality medical care (19).

T stage characterizes the primary tumor based on factors such as basal diameter, tumor thickness, and the presence or absence of extraocular extension. Our study found that as T stage increases, survival rates progressively decrease, with tumor size inversely correlated with patient survival. Higher T stages are associated with greater tumor thickness and diameter, which elevate the risk of retinal and Bruch's membrane rupture (20). Shields et al. demonstrated that each 1 mm increase in tumor thickness corresponded to a 5% increase in metastasis risk, ultimately leading to higher mortality rates (21).

Although surgery has traditionally been the primary treatment for choroidal melanoma, our study indicates that radiotherapy may provide superior survival outcomes, particularly for early-stage disease. Enucleation effectively removes the primary tumor; however, excessive manipulation or injury during surgery can increase the risk of tumor cell dissemination into the bloodstream and orbital tissues, potentially accelerating distant metastasis (22). Experimental studies have demonstrated that surgical trauma activates pro-inflammatory cytokines (e.g., IL-6, TNF-α) and matrix metalloproteinases (MMPs), promoting tumor cell migration and invasion. For instance, Beasley et al. (23) found that instrumental manipulation during ocular melanoma surgery significantly increased circulating tumor cells in peripheral blood, correlating with a higher risk of distant metastasis. In contrast, radiotherapy, particularly I-125 brachytherapy, minimizes physical disruption to the lesion while preserving ocular function and reducing systemic complications (7). This approach achieves effective tumor control and reduces the likelihood of iatrogenic tumor spread. Stalhammar demonstrated that I-125 brachytherapy for choroidal melanoma achieved a 10-year local control rate of 85%–90%, with minimal risk of extraocular extension or systemic dissemination (24). Radiation induces DNA double-strand breaks, activating the cGAS-STING pathway, promoting type I interferon release, and enhancing tumor antigen presentation (25). Additionally, radiation-induced DNA damage can trigger immunogenic cell death, potentially enhancing systemic anti-tumor immune responses. This systemic immune activation may partially explain the survival advantage observed in the radiotherapy group, whereas surgical trauma, through the release of pro-metastatic factors such as IL-6, may counteract these benefits (26, 27).

Despite the fact that traditional treatment modalities such as surgery and radiotherapy remain the cornerstone of choroidal melanoma management, recent advancements in emerging therapies have expanded treatment options for patients, particularly those with small- to medium-sized tumors. Transpupillary Thermotherapy (TTT) has emerged as a promising minimally invasive approach, utilizing laser energy delivered through the pupil to induce tumor vessel occlusion and subsequent necrosis (28). In addition to TTT, photodynamic therapy (PDT) has demonstrated significant efficacy in certain cases, especially for amelanotic choroidal melanoma (29). By intravenously administering a photosensitizing agent that accumulates in tumor tissue, PDT triggers the production of reactive oxygen species in the tumor microenvironment upon activation by light of a specific wavelength, leading to tumor cell destruction. In recent years, immunotherapy and targeted therapy have also rapidly advanced, offering novel therapeutic strategies for choroidal melanoma. Anti-CTLA-4 antibodies, such as ipilimumab, enhance T-cell activation and proliferation by blocking the interaction between CTLA-4 and its ligands CD80/86, thereby boosting the immune response against tumors (30). Studies have shown that ipilimumab significantly prolongs progression-free survival and overall survival in patients with advanced melanoma while also improving their quality of life (31). Furthermore, the combination of ipilimumab with chemotherapy or other immunotherapeutic agents, such as PD-1 inhibitors, may yield synergistic effects, further enhancing treatment outcomes. Targeted therapy focuses on specific molecular targets, such as BRAF, NRAS, c-KIT, and GNAQ/GNA11 gene mutations, which influence critical signaling pathways. The advantage of targeted therapy lies in its precision and the ability to minimize damage to normal cells, thereby reducing side effects (32, 33).

This study has several limitations. First, geographic variability and potential bias are present in the patient information recorded in the SEER registry. Second, the lack of detailed surgical procedure data in the SEER database precluded stratified analysis based on surgical approaches, thereby limiting the comparison of survival rates between different surgical methods and radiotherapy. Third, given the absence of specific codes for biopsy in the SEER database, we recommend that future studies utilize multi-center clinical data to elucidate the independent impact of biopsy on prognosis. Lastly, because of the inherent limitations of retrospective studies, the findings are inevitably biased, and further prospective studies are needed to confirm these results.

In conclusion, the choice of treatment for choroidal melanoma requires a comprehensive evaluation and individualized approaches. Key factors to consider include tumor size and morphology, tumor stage, patient age, overall health status, and treatment preferences. Through statistical analysis and data validation, this study supports the use of radiotherapy as a primary treatment modality for choroidal melanoma, particularly in early-stage disease. These findings hold significant implications for clinical practice.

## Conclusions

Among patients diagnosed with choroidal melanoma, those who underwent radiotherapy alone exhibited significantly higher overall survival (OS) and choroidal melanoma-specific survival rates compared to those who received surgical intervention alone. However, for patients with advanced disease or evidence of metastatic spread, the individualization of treatment regimens remains critically important.

## Data Availability

Publicly available datasets were analyzed in this study. This data can be found here: https://seer.cancer.gov/.
