# Correction: Radiotherapy has a survival advantage over surgery in patients with choroidal melanoma: a retrospective cohort study of 6,871 patients

**DOI:** 10.3389/fsurg.2025.1660922

**Published:** 2025-08-01

**Authors:** Yifan Wu, Lu Shi, Zhiqiang Ye, Yi Zhou, Feiran Wang, Yulan Zhang

**Affiliations:** ^1^ Department of Ophthalmology, The Second Affiliated Hospital, Jiangxi Medical College, Nanchang University, Nanchang, Jiangxi, China; ^2^ Department of Ophthalmology, The Second Affiliated Hospital, Nanchang University, Nanchang, Jiangxi, China; ^3^ Department of General Practice, The Second Affiliated Hospital, Jiangxi Medical College, Nanchang University, Nanchang, Jiangxi, China

**Keywords:** choroidal melanoma, uveal melanoma, mortality, SEER database, treatment

**Text correction**

In the published article, there was an error. No explanation was given in the statistical analysis section for the choice of TNM staging system over tumor size alone as the analysis method.

A correction has been made to **[Materials and methods]**, *[Statistical analysis***]**. The sentence previously stated:

“[Cox proportional hazards regression analysis was employed to examine the effects of age, race, sex, year of diagnosis, laterality, histology, T stage, N stage, M stage, treatment, and marital status on all-cause mortality and choroidal melanoma-specific mortality in patients with choroidal melanoma.]”

The corrected sentence appears below:

“[Cox proportional hazards regression analysis was employed to examine the effects of age, race, sex, year of diagnosis, laterality, histology, treatment, marital status, T stage, N stage, and M stage on all-cause mortality and choroidal melanoma-specific mortality in patients with choroidal melanoma. (The TNM staging system characterizes tumor biology through standardized evaluation of three components: primary tumor dimensions, regional lymph node status, and presence of distant metastases.)]”

The original article has been updated.

